# An overview of the British Columbia Glomerulonephritis network and registry: integrating knowledge generation and translation within a single framework

**DOI:** 10.1186/1471-2369-14-236

**Published:** 2013-10-29

**Authors:** Sean Barbour, Monica Beaulieu, Jagbir Gill, Ognjenka Djurdjev, Heather Reich, Adeera Levin

**Affiliations:** 1 Division of Nephrology, University of British Columbia, Vancouver, BC, Canada; 2 BC Provincial Renal Agency, Vancouver, BC, Canada; 3 Centre for Health Evaluation and Outcomes Research, St. Paul’s Hospital, Vancouver, BC, Canada; 4 Division of Nephrology, University of Toronto, Toronto, BC, Canada

**Keywords:** Glomerulonephritis, Registry, Database, Health services, Knowledge translation, British Columbia, Canada

## Abstract

**Background:**

Glomerulonephritis (GN) is a group of rare kidney diseases with a substantial health burden and high risk of progression to end-stage renal disease. Research in GN has been limited by poor availability of large comprehensive registries. Substantial variations in access to and administration of treatment and outcomes in GN have been described. Leveraging provincial resources and existing infrastructure, the British Columbia (BC) GN Network is an initiative which serves to combine research and clinical care objectives. The goal of the BC GN Network is to coordinate and improve health care, including robust data capture, on all patients with GN in BC, a Canadian province of over 4.6 million people. This provincial initiative will serve as a model for Canadian or other national and international endeavours.

**Description:**

The BC Provincial Renal Agency (BCPRA) is the provincial governmental agency responsible for health delivery for all kidney patients in BC. The BC GN Network has been created by the BCPRA to ensure high quality and equitable access to care for all patients with GN and is a platform for evidence based clinical care programs and associated health policy. All patients with biopsy-proven GN are registered at the time of kidney biopsy into the BCPRA provincial database of kidney disease patients, forming the BC GN Registry. Thereafter, all laboratory results and renal related outcomes are captured automatically. Histology data and core clinical variables are entered into the database. Additional linkages between the GN Registry and administrative databases ensure robust capture of medications, hospital admissions, health care utilization, comorbidities, cancer and cardiac outcomes, and vital statistics.

**Conclusions:**

The BC GN Network and Registry is a unique model in that it combines robust data capture, data linkages, and health care delivery and evaluation into one integrated system. This model utilizes existing health infrastructure to prospectively capture population level data on patients with GN, producing a rich dataset capable of real-time identification and evaluation of GN health policy initiatives, of supporting observational cohort studies and health services research in GN, and of facilitating patient recruitment into GN clinical trials.

## Background

While glomerulonephritis (GN) is considered a group of rare diseases, with an incidence of 0.7-2.8 per 100000/year depending on the specific type of GN, collectively they may consume substantial health care resources [1,2]. The risk of progression to end-stage renal disease (ESRD) is high, between 20-50%, such that GN is the second most common cause of ESRD in Canada [3-9]. Further, there is additional morbidity and cost associated with the treatment of GN, related both to expensive immunosuppressive medications, and infectious, malignancy or other complications of treatment. Consequently, the burden of GN to the health care system is substantial.

Due to the rarity of GN, registries that sample large geographic regions, and thus large numbers of patients, are required for collection of sufficient data to evaluate and improve health policy and systems of care for GN patients, and to facilitate clinic outcomes research in glomerular diseases [1,10]. The infrequent nature of the disease contributes to the poor understanding of these conditions by individual clinicians who may be exposed to small numbers of patients. Similar to cancer registries, the potential benefits of central registration of GN patients into regional databases may include enhanced physician collaboration resulting in improved patient care [1]. The absence of such registries significantly limits the understanding of the natural history of GN and the recruitment of GN patients into clinical trials, and thus impedes research and results in a void of evidence-based treatment recommendations. While existing GN registries have substantially contributed to the advancement of GN research, they are often research-based databases limited in their recruitment scope, expensive and labour-intensive to maintain, and not well integrated into patient care. Given these limitations they are not likely to be sustainable in the long-term.

The British Columbia (BC) GN Network and Registry has been created to address the limitations of other registries by leveraging existing health services infrastructure and data capture to facilitate and coordinate the care of all patients with GN in BC, a Canadian province of over 4.6 million people with a universal health care system. The components of the BC GN Network include the creation of a formal GN Registry embedded within an existing provincial information system, and the creation of an advisory group of relevant stakeholders including physicians, researchers, pharmacists, nurses and analysts, with direct responsibility for the administration of the BC GN Network and accountability for its outcomes. Both the database and the advisory group utilize existing infrastructure and processes within a larger provincial kidney disease network.

### Overarching goals of the BC GN Network

1. To create a sustainable framework for knowledge translation, policy development and implementation of best practice management through the systematic collection and analysis of data

2. To identify, implement and evaluate clinical care programs and health policy initiatives that improve the care of patients with GN in BC in a financially sustainable fashion.

3. To promote clinical outcomes, health policy and health services research in the field of GN.

4. To ensure knowledge translation of such initiatives to key stakeholders, including patients and physicians.

### Specific goals of the BC GN Registry

1. To prospectively capture at the time of kidney biopsy all relevant clinical, laboratory, pathology and outcome data in GN patients.

2. To collate data regarding the incidence, prevalence, outcomes, and health care utilization of patients with GN.

3. To capture such data using a sustainable infrastructure that leverages existing administrative resources.

4. To ensure high quality data is available to identify, develop and evaluate new clinical care and health policy initiatives targeting patients with GN.

5. To provide a rich dataset capable of supporting clinical outcomes and health services research in the field of GN.

## Construction and content

### The provincial infrastructure for chronic kidney disease in BC

The BC Provincial Renal Agency (BCPRA) is a governmental organization that coordinates, monitors and funds the delivery of care to patients with kidney disease in BC (http://www.bcrenalagency.ca). The mandate of the BCPRA is to improve the quality of life and outcomes for patients with renal disease, and to promote a fiscally sustainable health care system. The BCPRA operates the Patient Records and Outcomes Management Information System (PROMIS), which is a provincial database of all patients with chronic kidney disease (CKD) in BC. PROMIS is an Oracle-based web-accessible database with over 10000 patient-records that was initiated in 2000 and captures patients with CKD, defined as evidence of kidney damage either with an estimated (e)GFR <60 ml/min/1.73 m^2^, or structural abnormalities on imaging or kidney biopsy. Registration in PROMIS is mandatory for funding of CKD medications (such as erythropoietin stimulating agents, phosphate binders or vitamin D analogues), access to multidisciplinary care teams, or to receive renal replacement therapy including dialysis and transplantation. As such, PROMIS serves as a central source of data for all patients seen by nephrologists or kidney care teams in BC. PROMIS interfaces with all laboratory systems in the province, which permits automatic data capture and upload into a central database. Clinical data captured in PROMIS includes comorbidities, medications, blood pressure, weight and height. Funding is in place for data capture and formal data quality checks.

While technically able to register any patient with GN, PROMIS was not specifically designed as a database for patients with glomerular diseases who often have normal eGFR. Review of existing biopsy databases identified that approximately 66% of patients with IgA nephropathy and 25% of those with pauci-immune glomerulonephritis are not registered in PROMIS, which may be related to preserved renal function in a subgroup of patients, or a competing risk of death preventing registration [11]. Further, GN patients in BC have historically been registered late in the course of their disease when renal function has significantly declined, with little historical data available between the time of kidney biopsy and the development of compromised kidney function requiring access to “CKD resources”. The BC GN Network and Registry utilizes the existing infrastructure at both the BCPRA and PROMIS to capture data on patients with biopsy-proven GN as of the time of kidney biopsy.

### The structure of the BC GN Network

The BC GN Network is an initiative of the BCPRA that coordinates and evaluates the care of patients with GN in BC, with 5 geographical health authorities with more than 70 nephrologists. The BC GN Network has an established set of terms of reference (see Additional file 1), and is comprised of a formal steering committee of stakeholders interested in GN, including physicians, researchers, pharmacists, nurses and data analysts. To maintain integration within the larger provincial renal community, the steering committee has reporting obligations to the nephrology research unit within the University of British Columbia and to administrative committees within the BCPRA, including the Executive Committee, the Medical Advisory Committee, the Pharmacy and Formulary Committee, and the Information Management Council.

The BC GN Network focuses on several domains of GN-related health care as outlined in Table 1. This includes developing and evaluating health policy in GN, encouraging clinical and health services research in glomerular diseases, engaging key stakeholders of the renal community, and improving education and knowledge translation to end users. In addition, a significant activity of the BC GN Network is the implementation and oversight of the BC GN Registry to collect data necessary to support the goals of the BC GN Network. Detailed information can be found on the website: http://www.bcrenalagency.ca/professionals/GNNetworkRegistry.

**Table 1:** Domains and activities of the BC GN Network with specific examples

**Domains of the BC GN network**	**Examples**
Developing and evaluating health care initiatives specific to GN patients	GN specialty multi-disciplinary clinics with telehealth outreach to all geographic regions in the province
	Standardized GN-specific immunosuppression orders and laboratory requisitions
	GN-specific laboratory and medication flow sheets
	Centralized funding for immunosuppression medications
Collection of data necessary to support the goals of the BC GN Network	BC GN Registry
	Regular reporting to the provincial renal community on the incidence, prevalence, outcomes and health care utilization of GN patients in BC
Encouraging research in the field of GN	Utilization of the BC GN Registry for health outcomes and health services research
	Facilitating recruitment of patients into GN clinical trials by identifying eligible patients in the BC GN Registry
Engaging patients, physicians and other members of the renal community in the development of GN-specific initiatives	BC GN Network Steering Committee has representation from multiple health care domains, including pharmacists, academic and community physicians, and database / analytic specialists
Education and knowledge translation to physicians, patients and other stakeholders	Provincial GN rounds
	Patient education tools, ex. medication information sheets
	Dissemination of information through the GN Network website: http://www.bcrenalagency.ca/professionals/GNNetworkRegistry

### The BC GN Registry

**Figure 1:**
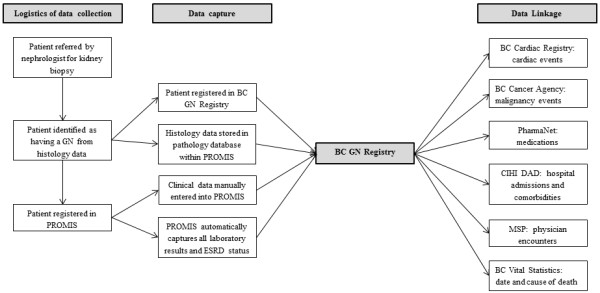
**The logistics of data capture for the BC GN Registry.** BCPRA = BC Provincial Renal Agency. CIHI DAD: Canadian Institute of Health Information Discharge Abstract Database. MSP = Medical Services Plan.

**Figure 2:**
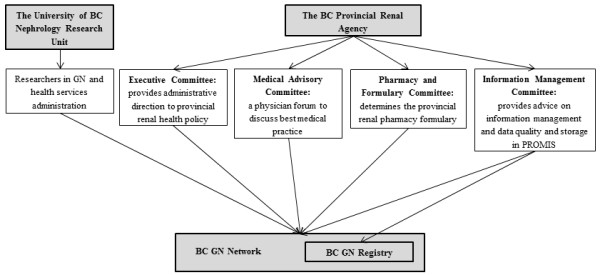
The structure of the BC GN Network and Registry with the University of British Columbia and the BC Provincial Renal Agency.

As of March 1^st^ 2013, the BC GN Registry prospectively captures data on all patients with GN in BC as of the time of kidney biopsy, with data storage in PROMIS. It is comprised of a combination of existing BCPRA resources, a new pathology database initiative, data extraction of key clinical variables, and the ability to link with administrative datasets to capture outcomes of interest (see Figure 1). The structure of the BC GN Network and Registry is shown in Figure 2.

#### Integration of provincial and regional pathology databases into PROMIS

All renal biopsies in BC are interpreted at two centres: the Royal Jubilee Hospital in Victoria, which processes all biopsies performed on Vancouver Island, and the BC Renal Pathology Laboratory based in Vancouver, which processes all biopsies in the remainder of the province. Prior to the 2013 launch of this initiative, each centre previously maintained independent locally managed pathology databases. These have been replaced with a single centralized pathology database within PROMIS containing key histologic variables from each biopsy performed (see Table 2). During the initial phase of the BC GN Network, histologic data from the pathology reports is being manually entered into PROMIS by trained data analysts. Development of a robust user-interface is ongoing and will permit pathologists to enter data directly at the time of biopsy interpretation, reducing duplication of data entry and ensuring seamless access to key data.

**Table 2:** Variables stored in PROMIS as part of the provincial pathology database, with representative examples

**Variables in pathology**	**Examples**
**database**	
Patient details	Name, date of birth, Personal Health Number, geographic location, sex
Biopsy details	Date of biopsy, native versus transplant biopsy, surgical case number
Primary and secondary diagnoses	As per pathologists’ discretion
Quantitative pathology details	Number of glomeruli
	Number of obsolete glomeruli
	Number of segmentally sclerotic glomeruli Number of glomeruli with crescents
	Severity of interstitial fibrosis and tubular atrophy
	Severity of arteriolar and arterial vascular disease
Immunofluorescence findings	0-3+ for each of IgG, IgM, IgA, C3 and C1q
Disease-specific classifications	IgA nephropathy: MEST score
	Transplant allograft: Banff classification
	Lupus nephritis: ISN/RPS classification
	FSGS: Columbia classification
	ANCA vasculitis: EUVAS classification

MEST = based on Oxford classification [12]. ISN/RPS = International Society of Nephrology / Renal Pathology Society. FSGS = focal segmental glomerulosclerosis.

#### Registration in the BC GN Registry

The inclusion criterion of the BC GN Registry is the diagnosis on renal biopsy of any glomerular disease found in Table 3. The diagnosis of GN results in automatic registration in PROMIS and the BC GN Registry, which triggers a set of activities that ensure consistent capture of essential data. Patients with other types of biopsy-proven renal diseases, such as diabetes, hypertensive nephrosclerosis or interstitial nephritis are not included in the GN registry. These patients are however captured in the provincial pathology database.

**Table 3:** List of native-kidney biopsy diagnoses that are used to identify patients eligible for registration in the BC GN Registry

**Histologic diagnoses enrolled in the BC GN registry**	
Alport’s disease	Idiopathic nodular glomerulosclerosis
Amyloidosis: AL	IgA nephropathy
Amyloidosis: AA	IgM nephropathy
Amyloidosis: other	Immunotactoid GN
Anti-GBM antibody (Goodpasture’s) GN	Light chain deposition disease
Autoimmune/connective tissue disease associated GN	Light and heavy chain deposition disease
Cryoglobulinemic GN	Lupus nephritis
C3 GN	Minimal change disease
C1q nephropathy	Membranoproliferative GN, complement mediated
Dense deposit disease	Membranoproliferative GN, IC mediated
Fabry’s disease	Membranous nephropathy
Fibrillary GN	Mesangial proliferative GN
Focal segment glomerulosclerosis	Pauci-immune (ANCA) GN
GN with monoclonal immunoglobulin deposits	Post-infectious GN
GN not otherwise specified	Proliferative GN
Heavy chain deposition disease	Thin basement membrane disease
Henoch-Schonlein purpura	Waldenstrom’s macroglobulinemia

This list may be updated in the future as histologic classifications change over time.

#### Anticipated incidence of GN in BC

A review of existing pathology databases permits an estimate of the anticipated yearly incidence of GN in BC. During the 12-year prior from 2000–2012, there were 3473 cases of the common glomerular diseases, with an average of 289 cases per year (see Table 4). The most frequent types of GN in BC are lupus nephritis (62 cases per year), IgA nephropathy (69 cases per year), and focal segmental glomeruloslerosis (70 cases per year).

**Table 4:** The number of cases of each type of GN in the BC pathology databases from 2000 to 2012

**GN type**	**Number of cases**
Minimal Change Disease	263
IgA Nephropathy	827
Focal Segmental Glomeruloslerosis	846
Membranous Nephropathy	418
Lupus Nephritis	738
ANCA Vasculitis	381

The average number of GN cases per year is 267.

#### Capture of full laboratory data

Because the BC GN Registry is embedded within PROMIS, once the patient is registered all laboratory results are automatically uploaded through established and sanctioned interfaces between PROMIS and provincial laboratory systems (see above). Relevant laboratory results for patients with glomerular diseases, such as proteinuria, albumin, cholesterol, creatinine, and autoimmune and infectious disease serology are captured at whatever frequency they are ordered by the primary physician.

As part of a province-wide eGFR reporting initiative, creatinine measurements at all laboratories in BC have been calibrated using an isotope dilution mass spectrometry (IDMS) reference standard and used in the IDMS traceable version of the MDRD formula [13]. As such, all creatinine measurements and eGFR estimates are standardized and directly comparable across laboratories.

#### Capture of clinical data

Date of birth, sex, and geographic location are required at the time of registration in PROMIS. Additional clinical data is captured from the primary nephrologists in accordance with best practices and existing technologies. The clinical variables that are collected are as follows: comorbidities, medications, blood pressure, height and weight. While these variables are not extensive, they represent a balance between feasibility and cost, with the minimum data required to accurately describe a cohort of patients with GN. The same data is collected for all CKD, ESRD, and kidney transplant patients as part of the provincial mandate of the BCPRA.

#### Capture of renal outcomes and mortality

There is complete capture of patient enrollment in CKD clinics, timing and type of ESRD modality, transplant outcomes and mortality as part of the provincial mandate and existing infrastructure. Only those patients who move out of province are lost to follow up. Therefore, these outcomes will be available for all patients in the BC GN Registry.

#### Data security and integrity

The BC GN Registry is contained within PROMIS, which is an administrative health care database that stores fully identified patient-level data and is managed by the BCPRA under regulation by the provincial government of BC. It is a fully encrypted Oracle-based database, with regular backup to both servers and magnetic tapes. All users have access only to data deemed necessary for their clinical and/or administrative positions, and require secure passwords for login. All successful and unsuccessful login attempts and data entry events are tracked. The security systems and practices for PROMIS are similar to those used for other governmental health care agencies in BC.

### Linkage of the BC GN Registry with other administrative databases

Each person in BC has a unique Personal Health Number (PHN), which is required for registration into PROMIS and is therefore captured by the BC GN Registry at the time of kidney biopsy. Using the PHN, the BC GN Registry has been linked to various other governmental administrative databases. Examples include the BC Cardiac Registry to identify angiographic or surgical cardiac interventions; the BC Cancer Agency to identify malignancy events; PharmaNet to capture all medications dispensed at out-patient pharmacies in the province; the Canadian Institute of Health Information Discharge Abstract Database to identify hospital admissions, discharge diagnoses and comorbidities; the Medical Services Plan to identify all visits to physicians; and the Vital Statistics Database for cause and date of death. This is shown in Figure 1. Processes and procedures for this linkage will be in accordance with all privacy legislation, and differ depending on the purpose of data usage.

## Utility and discussion

British Columbia is a Canadian province with over 4.6 million inhabitants and approximately 289 cases of GN per year. This represents a substantial incidence of patients who consume significant health care resources and are at increased risk of progression to ESRD. Prior to this initiative, as is the case in many other national and international jurisdictions, in BC there was no consistent formalized framework or data capture to inform the care of patients with GN. Thus, despite the large number of incident and prevalent GN patients, there has been little published data on short and long-term outcomes.

The BC GN Network and Registry represents a novel approach of integrating prospective data collection on GN patients into a provincial clinical care program for glomerular diseases, which will facilitate the development and evaluation of health policy and health services initiatives aimed at improving the outcomes of patients with GN. The BC GN Network is a provincial initiative that incorporates representation from BCPRA administrative bodies involved in health services delivery relevant to GN, one component of which is the GN Registry within PROMIS. This integration of data capture within the larger framework of GN health administration will allow for robust evaluation of health policy or clinical care programs developed by the BC GN Network. Indeed, this model has proven effective at improving the outcomes of all-cause CKD patients in BC. PROMIS was designed as a CKD administrative database to inform the renal-specific health care delivery of the BCPRA. It has been used to show that CKD patients managed in multi-disciplinary care clinics have better metabolic parameters and improved survival on dialysis compared to those managed by nephrologists [14]. Further, comprehensive data obtained from PROMIS has been used to identify substantial cost savings by managing CKD patients in a combined specialist clinic, describe provincial variability in testing for hepatitis B immune status in renal patients, and identify rare but severe complications from dialyzer membranes [15-17]. The model of integrating data collection into health care delivery has already proven effective in BC in all-cause CKD, and it is anticipated that similar results will be seen in patients with glomerular disease.

Combining the BC GN Registry with the health services delivery and administrative activities of the BCPRA has the additional benefit of improving the scope and reducing the cost of data capture for a rare disease such as GN. Existing GN registries have provided substantial insight into the natural history of glomerular diseases and have been invaluable in the advancement of GN research [18-22]. However, most GN registries have been research-based, limited in the geographic scope or type of patient recruitment, and have relied on substantial amounts of manual data collection [1]. By comparison, the BC GN Registry is unique in that it is integrated into the existing provincial CKD health delivery infrastructure and uses linkages to laboratory and administrative databases to minimize laborious data entry, improving data quality and quantity. This approach ensures the long-term feasibility of the registry and allows complete capture of relevant patient information throughout the geographically diverse province of BC. In the study of rare diseases such as GN, comprehensive data capture of large numbers of patients over long periods of time is viewed as essential.

Research in the field of GN has been substantially limited by the rarity of the disease and the poor availability and substantial cost of detailed registries with long-term follow-up that sample large source populations [1,23]. The BC GN Registry provides a feasible and sustainable solution that will produce a rich dataset with clinically relevant renal and mortality outcomes capable of supporting research in glomerular disease. Data collection leverages existing interfaces to automatically capture laboratory results and ESRD status, which limits manual data entry to only core elements. Further, the ability to link administrative datasets provides the opportunity to capture detailed outcome information as shown in Figure 1 without the need for laborious prospective data entry. The provincial scope of the BC GN Registry allows patient recruitment from the entire population of BC, thereby improving generalizability compared to research-based GN registries that are often subject to selection bias from focused enrollment of patients from tertiary referral centres. The universal health care system in Canada helps reduce some of the bias that results from differential access to care. This approach to data collection ensures that over time the BC GN Registry will have a sufficient number of cases with detailed data capture to support clinical outcomes and health services research in GN. Furthermore, a coordinated provincial strategy for identifying and following patients with GN will facilitate recruitment into prospective clinical trials. This unique approach can serve as a model for other provinces in Canada, and as a nidus for Canadian GN collaboration.

There are several limitations to the data collected in the BC GN Registry. Firstly, in an attempt to improve the feasibility of data collection and the geographic distribution of patient enrolment, decisions were made regarding the need for a limited set of core clinical variables. This can be viewed as a relative limitation, as additional linkages with administrative datasets will be able to supplement prospectively collected data. Specific research questions can always be supplemented by review of individual patient records as required. Further, the BC GN Registry only enrolls patients with biopsy-proven disease, and would not necessarily be generalizable to patients with presumed GN that has not been confirmed on kidney biopsy. However, standard nephrology practice in Canada is to biopsy adult patients with clinically relevant proteinuria, especially in the context of declining renal function [24]. As such, patients with GN who do not undergo a biopsy would be expected to have mild disease with a favourable long-term prognosis. Further, the reliance on kidney biopsy as the point of identification and registration may complicate the ability to differentiate primary versus secondary disease in future research analyses. This will be partially mitigated by complete capture of laboratory data (such as autoimmune and viral serology) that are used to identify secondary causes of GN. Finally, there is a need for regular data validation methods so as to ensure correct classification and data entry. Pragmatic and robust processes of data validation are being developed and will be the subject of a future publication, once a sufficient sample of patient records is available.

## Conclusion

The BC GN Network is a unique approach to improving the understanding and care of patients with GN, a rare but important subset of patients with CKD. The BC GN Registry constitutes the core of the network, and is a comprehensive prospective database of patients with GN in BC. The existence of the registry will support future clinical outcomes and health services research in glomerular disease. The GN network utilizes a novel approach to data collection in GN by being integrated into the existing CKD health services infrastructure and by including automatic capture of data through linkages to laboratories and administrative databases. This reduces laborious manual data entry, thereby broadening the geographic region from which patients can be recruited. The BC GN Network is a model of integrating knowledge acquisition, interpretation and application in real time.

## Availability and requirements

The BC GN Registry data is contained in PROMIS, which is under regulation by governmental agencies in BC (the BCPRA and the Provincial Health Services Authority). Details about accessing PROMIS data for research purposes can be found at the BCPRA website (http://www.bcrenalagency.ca). Anyone interested in accessing PROMIS data for non-commercial research purposes must complete a data application request (which will be vetted by the BCPRA) and secure ethics approval through the University of British Columbia. Linkage of PROMIS data to other administrative databases may require additional approval from appropriate data stewards.

## Competing interest

The authors declare that they have no competing interests.

## Authors’ contributions

SB, MB, JG and AL have been involved in the development and implementation of the BC GN Registry and the BC GN Network, including the logistics and processes around patient registration and data capture. All authors contributed to manuscript writing, editing and submission. All authors read and approved the final manuscript.

## Pre-publication history

The pre-publication history for this paper can be accessed here:

http://www.biomedcentral.com/1471-2369/14/236/prepub

## Supplementary Material

Additional file 1 BC Glomerulonephritis (GN) Network Steering Committee. Click here for file
