# Supplementary material for: An overview of the British Columbia Glomerulonephritis network and registry: integrating knowledge generation and translation within a single framework
Source: BMC Nephrol. 2013 Oct 29;14:236. doi: 10.1186/1471-2369-14-236 (PMC3871721; doi:10.1186/1471-2369-14-236)
Supplement: Additional file 1 — BC Glomerulonephritis (GN) Network Steering Committee. [file 1471-2369-14-236-S1.pdf]

BC Glomerulonephritis (GN) Network Steering Committee

Terms of Reference (May 2013)

Background

The BC GN Network is an initiative of the BPRA whose overall goal is to identify, implement and evaluate health care programs and health policy initiatives that target patients with glomerulonephritis (GN), and to ensure communication of such activities to relevant stakeholders. The BC GN Network falls within the mandate of the current BCPRA infrastructure because patients with GN have been identified as unique in that they represent a rare and high risk subpopulation of CKD patients who will benefit from provincial infrastructure support.

The BC GN Network has a number of objectives:

- To facilitate evidence-based clinical management and treatment of glomerular diseases for patients throughout the province through development and utilization of standardized tools:
    - eg. prescription and blood work requisitions; laboratory and medication flow sheets in PROMIS
    - eg. ensuring equal access and funding for immunosuppressive agents
    - eg. pre-conception counseling for high-risk women with GN, with a coordinated management strategy between nephrology, obstetrics and maternal-fetal medicine for pregnant women with GN
- To develop and implement a set of knowledge translation activities for physicians, patients and other stakeholders:
    - e.g. provincial GN-specific rounds, email updates of the BC GN Network initiatives, web-based patient focused information sheets
- To ensure accountability and transparency regarding outcomes of patients with GN:
    - e.g. describe the incidence, prevalence, outcomes and health utilization of GN patients in BC
- To promote research in glomerular diseases through access to:
    - Clinical trials
    - Investigational medications
    - Observational cohort studies

The BC GN Network is comprised of the following:

- Health care providers with an interest in GN, including physicians, pharmacists, IT and statistical specialists and others, who are represented by the Steering Committee
- Health services specific to patients with GN
    - eg. GN clinic, standardized laboratory and prescription templates
- Educational initiatives regarding GN targeting key stakeholders

   - eg. physician rounds, patient medication information sheets
- Data capture and reporting on patients with GN, using the BC GN Registry

A fundamental activity of the BC GN Network is to integrate information, research and clinical activities, so that systematic evaluation of GN-related initiatives is possible in real time. Specifically, the BC GN Network will describe the number of GN cases in BC, clinical outcomes, and health utilization. The BC GN Registry, embedded within existing BCPRA information systems (PROMIS), has been established to facilitate data capture. As of March 1, 2013, prospective data on patients with GN in BC is being captured as of the time of kidney biopsy. This unique dataset will be used to support the goals and activities of the BC GN Network.

Terms of Reference (May 2013)

Category	Description
**1. Purpose**	To facilitate the identification, implementation and evaluation of clinical care programs and health policy initiatives that improve the care of patients with GN in BC in a financially sustainable fashion.<br><br>To ensure knowledge translation of such initiatives to key stakeholders, including patients and physicians.<br><br>To facilitate prospective capture of important information in the BC GN Registry (e.g. clinical, laboratory, pathology and outcome data) which is necessary to support the goals of the BC GN Network, and to promote data capture through a sustainable infrastructure that leverages existing resources.<br><br>To ensure awareness and promotion of information generated from the BC GN Network, and to promote research in the field of GN.<br><br>To monitor and provide information to the provincial renal community regarding the incidence, prevalence, outcomes, and health care utilization of patients with GN.
**2. Responsibilities**	To provide guidance and oversight to provincial GN initiatives that relate to the above stated goals, and to ensure implementation of such initiatives.<br><br>To appoint, as necessary, work groups as the Steering Committee deems necessary to address specific initiatives related to the above stated goals.<br><br>To receive, consider and act upon recommendations related to GN that are received from the above stated sub-committees, other committees within the BCPRA, and other health care providers outside of the BCPRA.<br><br>To provide advice regarding the capture and utilization of data for patients with GN in BC.
**3. Deliverables**	To provide regular communication with the provincial nephrology community regarding the activities of the BC GN Network.<br><br>To provide regular updates to the BCPRA executive regarding the activities of the BC GN Network.<br><br>To develop mechanisms by which regular data presentation and review can be conducted with the provincial nephrology community regarding the incidence, prevalence, health utilization and outcomes of patients with GN in BC.
**4. Composition and appointment process**	The BC GN Network Steering Committee can consist of at least the following (which do not need to be mutually exclusive):<br>1. A member from each of the following BCPRA committees: MAC, Pharmacy and Formulary, KCAC

	2. The BCPRA executive director<br>3. A member from VCH, FHA, VIHA, Interior HA, NH, and BC Children's<br><br>4. A representative from PROMIS<br>5. 1-2 members experienced in GN research and/or other research-related fields<br>6. The BC GN Network and Registry Medical Lead<br>7. Representatives from Rheumatology and Obstetric Medicine<br>8. Representation from laboratory medicine and Pathology
**5. Chair and reporting relationships**	The Medical Lead of the BC GN Network and Registry will be Chair, reporting to the MAC and the Administrative Executive quarterly<br><br>Review of duration of term and reporting relationships will be reviewed after 24 months of initial creation of the Steering Committee, in order to evaluate functionality of the structure as created and modify as needed.
**6a. Duties of the chair**	Organize and chair BC GN Network Steering Committee meetings every 6 months, or as required.
**6b. Duties of the committee members**	To act as representatives of their respective health authority, BCPRA committee or other appropriate specialty groups; and to communicate with such groups regarding the activities of the Steering Committee.
**7. Meetings**	The BC GN Network Steering Committee shall meet at least every 6 months, or more frequently as needed, either in person or by teleconference.<br><br>Notice of such meetings shall be initiated by the Chair.<br><br>Minutes of each meeting will be kept and circulated.<br><br>Each voting member of the committee shall attend in person or by phone at least 2/3 of all meetings. The BCPRA executive director in consultation with the Chair may remove any voting member who does not meet this attendance requirement.<br><br>Work group meetings may be more frequent.
**8. Quorum**	A quorum shall be defined as the majority of the voting members present at a meeting within 20 minutes of the scheduled meeting start, failure to meet quorum shall result in deferral of the meeting.
**9. Voting**	Each voting member has one vote, and must be present in person or by teleconference to vote, no votes by proxy will be allowed. The Chair is allowed a second vote in the event of a tie. All initiatives must pass by a majority of votes. The BCPRA Executive Director will not be a voting member.
**10. Remuneration**	There will be no remuneration for membership in the committee.

**11. Communications**	Members of the Steering committee shall represent their health authorities, BCPRA committees or other specialty groups in issues related to GN. Members shall communicate regularly with their respective groups regarding activities of the steering committee.
**12. BCPRA Support**	The BCPRA administrative support will ensure minutes and communications are distributed to members in a timely fashion, and will facilitate the coordination and operation of the Steering Committee activities as needed under the direction of the Chair.
